# Supplementary material for: Repurposing of the antimalarial agent tafenoquine to combat MRSA
Source: mSystems. 2023 Dec 4;8(6):e01026-23. doi: 10.1128/msystems.01026-23 (PMC10734505; doi:10.1128/msystems.01026-23)
Supplement: Supplemental material — Table S1, Figure S1, and supplemental movie legends. [file msystems.01026-23-s0001.docx]

**Repurposing of the anti-malarial agent tafenoquine to combat MRSA**

Yang Yifan^1,#^, She Pengfei^1,#^, Li Linhui^1^, Li Yimin^1^, Liu Shasha^1^, Li Zehao^1^, Zhou Linying^2^, Wu Yong^2,^*

1. Department of Laboratory Medicine, The Third Xiangya Hospital of Central South University, Changsha 410005, China.

2. Department of Laboratory Medicine, The Affiliated Changsha Hospital of Xiangya School of Medicine, Central South University, Changsha, China.

#: **Equal contribution and first authorship**: Yang Yifan and She Pengfei contributed equally to this work and share first authorship.

*** Correspondence:**

Corresponding Author: Wu Yong.

E-mail: [wuyong_zn@csu.edu.cn](mailto:wuyong_zn@csu.edu.cn)

**Tables S1.** The value of MICs by TAF in the presence/absence of potential competitors against *S. aureus* ATCC 43300.

**Supplementary Figure S1.** *In vivo* toxicity of TAF. (A) Time-survival of mice (n= 6 mice/group) after treated with TAF. (B-H) Quantification of organ biomarkers after TAF treatment for 24 h. (B) Renal function evaluated by blood urea nitrogen (BUN). (C) Myocardial function evaluated by creatine kinase (CK) quantification. Hematological parameters analysis included (D) white blood cell (WBC) counting, (E) Neutrophil percentage (N%), (F) platelet (PLT) counting, and (G) red blood cell (RBC) counting. (H) H&E staining of myocardium, liver, spleen, lung, and kidney. (B-G) Data are presented as means ± SD and analyzed by Student's t-test.

**Supplementary** **Movie S1.** MD simulation process of TAF interaction with 7DOPC/3DOPG phospholipid membranes.

**Supplementary Movie S2.** MD simulation process of TAF interaction with 7POPC/3cholesterol phospholipid bilayer.

**Tables S1.** The value of MICs by TAF in the presence/absence of potential competitors against *S. aureus* ATCC 43300.

| Chemicals | MICs (μg/mL) |
| --- | --- |
| TAF | 8 |
| GSH | >128 |
| TAF+7.5 mM GSH | 32 |
| PGN | >128 |
| TAF+40 μg/mL PGN | 8 |

**Figure S1**
